# Effect of Al–5Ti–C Master Alloy on the Microstructure and Mechanical Properties of Hypereutectic Al–20%Si Alloy

**DOI:** 10.3390/ma7021188

**Published:** 2014-02-14

**Authors:** Wanwu Ding, Tiandong Xia, Wenjun Zhao, Yangtao Xu

**Affiliations:** School of Materials Science and Engineering, Lanzhou University of Technology, Lanzhou 730050, Gansu, China; E-Mails: dingww@lut.cn (W.D.); zhaowj@lut.cn (W.Z.); xuyat@sina.com (Y.X.)

**Keywords:** Al–5Ti–C master alloy, hypereutectic Al–20%Si alloy, modification, primary Si, eutectic Si, mechanical properties

## Abstract

Al–5Ti–C master alloy was prepared and used to modify hypereutectic Al–20%Si alloy. The microstructure evolution and mechanical properties of hypereutectic Al–20%Si alloy with Al–5Ti–C master alloy additions (0, 0.4, 0.6, 1.0, 1.6 and 2.0 wt%) were investigated. The results show that, Al–5Ti–C master alloy (0.6 wt%, 10 min) can significantly refine both eutectic and primary Si of hypereutectic Al–20%Si alloy. The morphology of the primary Si crystals was significantly refined from a coarse polygonal and star-like shape to a fine polyhedral shape and the grain size of the primary Si was refined from roughly 90–120 μm to 20–50 μm. The eutectic Si phases were modified from a coarse platelet-like/needle-like structure to a fine fibrous structure with discrete particles. The Al–5Ti–C master alloy (0.6 wt%, 30 min) still has a good refinement effect. The ultimate tensile strength (UTS), elongation (El) and Brinell hardness (HB) of Al–20%Si alloy modified by the Al–5Ti–C master alloy (0.6 wt%, 10 min) increased by roughly 65%, 70% and 51%, respectively, due to decreasing the size and changing the morphology on the primary and eutectic Si crystals. The change in mechanical properties corresponds to evolution of the microstructure.

## 1. Introduction

Hypereutectic Al–Si alloys have been widely investigated because of their excellent properties, which include excellent wear and corrosion resistance, high temperature strength, low coefficient of thermal expansion, good cast performance, and high specific strength [1–3]. Therefore, hypereutectic Al–Si alloys are widely used in aeronautic, astronautic, and automobile industries. It has been documented extensively that the microstructure of hypereutectic Al–Si alloys, prepared by conventional casting routines, usually consist of a coarse primary silicon phase in a fibrous eutectic matrix [4–6]. The brittleness of the coarse Si crystals (both eutectic and primary silicon) is the main reason responsible for the poor properties of Al–Si alloys because coarse silicon crystals lead to premature crack initiation and fracture in tension [7,8]. In order to refine the primary silicon, many methods have been carried out, such as high-pressure casting, the rapid solidification technique [9], and melt overheating treatment [10–12]. However, these processes are complex and difficult to control. The desired properties cannot be obtained or may even become worse. Microstructure control using minor element addition is the most popular method due to its simplicity and effectiveness. Phosphor is the most effective refinement element of primary Si in hypereutectic Al–Si alloy [13,14]. The size of primary Si particles can be refined to 20–30 μm by adding minor amounts of phosphor. Because the phosphor has no modification effect on the eutectic Si, the eutectic Si can still keep the large needle shape. Recently, attention has been given to the complex modification of primary and eutectic Si in order to significantly enhance the mechanical properties of hypereutectic Al–Si alloys. It has been documented that rare earth (RE) metal can modify the eutectic Si in hypereutectic Al–Si alloys [15,16]. The refinement of primary Si crystals was also obtained with the addition of rare earth elements [17,18].

In recent years, Al–Ti–B and Al–Ti–C master alloys have been found to be good refiners for Al and its alloys [19–21]. Compared with TiB_2_ in Al–Ti–B master alloys, TiC particles in Al–Ti–C grain refiner as the heterogeneous nucleation core have a smaller aggregation tendency which is not affected by such elements as Zr, Cr, Mn and V. Therefore, more attention has been paid to the preparation, microstructure and performance of Al–Ti–C master alloys [19,22,23]. Recently, Sagstad [24] synthesized Al–Ti–B–Sr master alloys, combining grain refiners and modifiers together. It has also been reported that B and Sr have an adverse effect on grain refinement and modification because of the formation of a SrB_6_ compound [25], resulting in a mutual poisoning effect. Zhao [26] prepared Al–Ti–C–Sr master alloys and reported that satisfactory grain refining and modifying effects were obtained by the addition of Al–Ti–C–Sr alloys (0.5 wt%) to the A356 alloy. However, little has been reported on the microstructure and mechanical properties of hypereutectic Al–20%Si alloy with the addition of Al–Ti–C master alloy. In this study, Al–5Ti–C master alloy was prepared and adopted to modify hypereutectic Al–20%Si alloy with the aim of investigating the effect of Al–5Ti–C master alloy on the microstructure of hypereutectic Al–20%Si alloy. At the same time, the mechanical properties and hardness of the hypereutectic Al–20%Si alloy were also studied.

## 2. Results and Discussion

### 2.1. Microstructures of Al–5Ti–C Alloy

**Figure 1.**
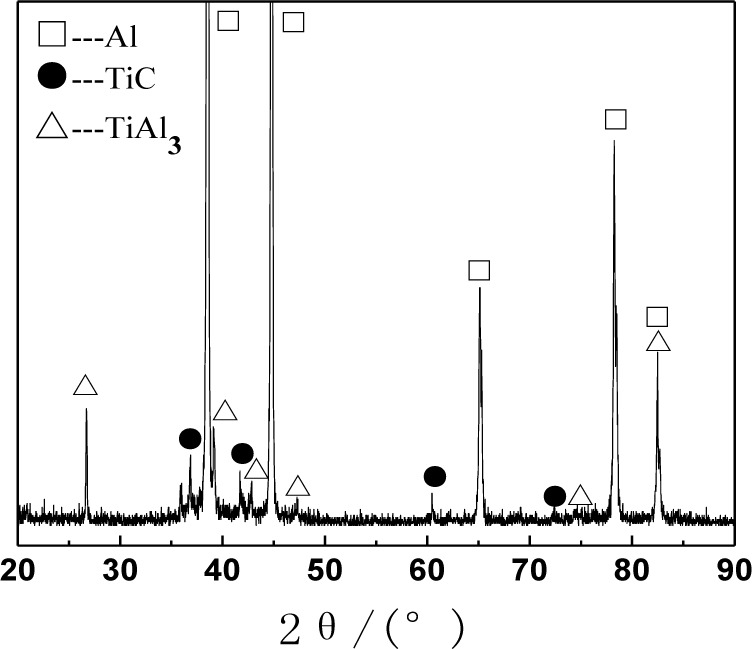
XRD pattern of Al–5Ti–C master alloy.

**Figure 2.**
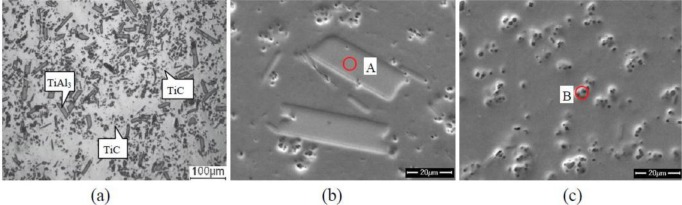
Microstructures of Al–5Ti–C alloy: (**a**) optical microscopy (OM) image; (**b**) scanning electron microscopy (SEM) image of TiAl_3_; and (**c**) SEM image of TiC.

Figure 1 shows the XRD (X-ray diffraction) pattern of the Al–5Ti–C alloys. As can be seen from Figure 1, Al–5Ti–C alloy is composed of Al, TiAl_3_ and TiC. Figure 2a shows the OM picture of the Al–5Ti–C alloy. As we can see from Figure 2a, on the Al substrate of Al–5Ti–C alloy a large number of strip-like or lump-like substances are uniformly distributed with a size of roughly 20–55 μm in length and 8–12 μm in width as well as small black particles. Figure 2b,c show the SEM image of the strip-like substances and that of the small black particles. Table 1 shows the analysis result of the energy spectrum of the chemical composition of point A in lump-like substances and point B in small particles in Figure 2. From Table 1, we can see that at point A in the strip-like substances, the molar mass fraction of element Al is 69.72%, the molar mass fraction of element Ti is 21.85%, and the molar mass ratio between element Al and element Ti is 3.19. At point B in the small particles, the molar mass fraction of element C is 27.98%, the molar mass fraction of element Ti is 25.52%, and the molar mass ratio between element C and element Ti is 1.1. According to the analysis results of the XRD pattern of Al–5Ti–C alloy, we can see that the strip-like substances in Figure 2b are TiAl_3_, and the small black particles in Figure 2c are TiC. From the above analysis, we know that in the Al–5Ti–C alloys used in the experiment a large number of strip-like or lump-like TiAl_3_ and TiC particles are contained, distributed in a dispersed and uniform way.

### 2.2. Microstructure Studies of Modified and Unmodified Al–20%Si Alloys

**Figure 3.**
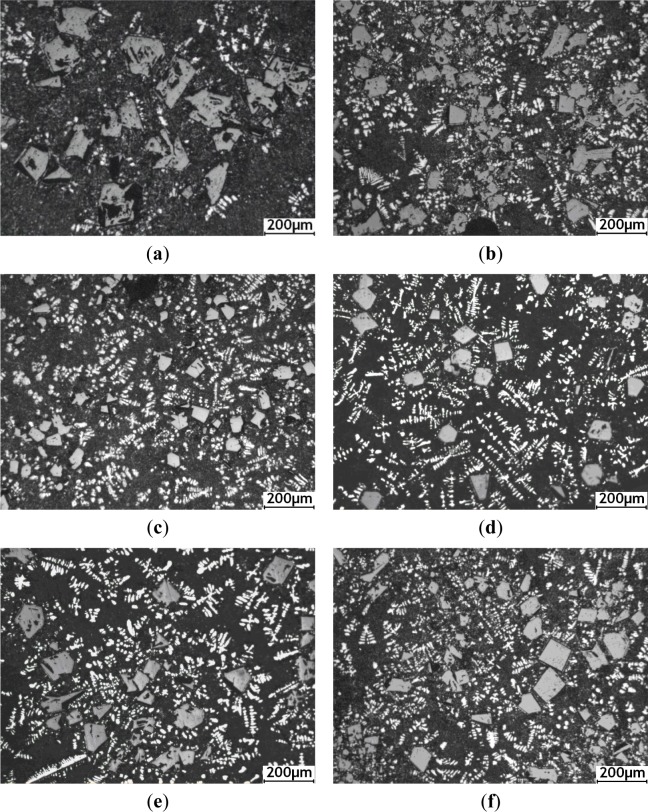
Optical micrographs of primary Si crystals in hypereutectic Al–20%Si alloy: (**a**) unmodified; (**b**) 0.4 wt% Al–5Ti–C; (**c**) 0.6 wt% Al–5Ti–C; (**d**) 1.0 wt% Al–5Ti–C; (**e**) 1.6 wt% Al–5Ti–C; and (**f**) 2.0 wt% Al–5Ti–C.

Figure 3 shows the optical micrographs of hypereutectic Al–20%Si alloy with various concentrations of Al–5Ti–C alloy, which demonstrates a substantial difference in microstructure of the size and morphology of the primary Si crystals. Figure 3a shows the optical micrograph of hypereutectic Al–20%Si alloy without addition of Al–5Ti–C alloy. It is clearly seen from Figure 3a that the primary Si crystals are present in the form of coarse polygonal shape and star-like shapes, and the average size of primary Si crystals is up to roughly 104 μm. Figure 3b presents the microstructure of Al–20%Si alloy inoculated with 0.4% Al–5Ti–C alloy. It is obvious that the coarse star-shaped primary Si phase is completely absent, and the average size of primary Si decreases to less than roughly 54 μm. Figure 3c shows the microstructure of Al–20%Si alloy containing Al–5Ti–C alloy, at levels of 0.6%. It is obvious that the edges and angles of most of the primary Si are passivated, and the average size of the primary Si further decreases to roughly 39 μm, with fine primary Si homogeneously distributed in the (α-Al + Si) eutectic matrix. However, when the addition of Al–5Ti–C alloy was further increased to 1.0%, 1.6% and 2.0%, the average size of primary Si crystals increased to roughly 50, 59 and 66 μm, respectively, as shown in Figure 3d–f.

**Figure 4.**
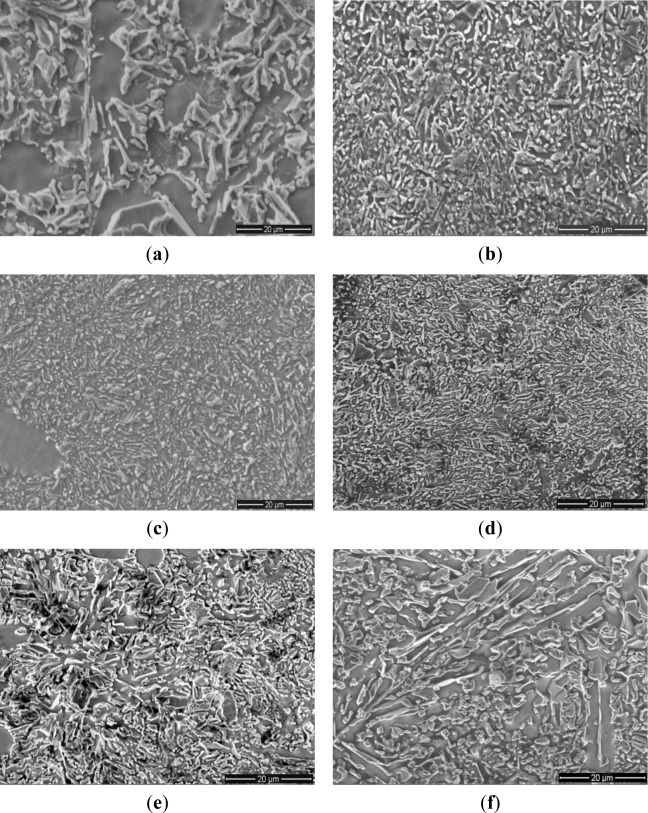
SEM images of eutectic Si crystals in hypereutectic Al–20%Si alloy: **(a**) unmodified; (**b**) 0.4 wt% Al–5Ti–C; (**c**) 0.6 wt% Al–5Ti–C; (**d**) 1.0 wt% Al–5Ti–C; (**e**) 1.6 wt% Al–5Ti–C; and (**f**) 2.0 wt% Al–5Ti–C.

The effect of Al–5Ti–C alloy on the morphology of eutectic Si is shown in SEM micrographs in Figure 4. Figure 4a shows the SEM micrograph of the eutectic Si structure in Al–20%Si alloy without addition of Al–5Ti–C alloy, which reveals coarse needles and platelets of eutectic Si in the metal matrix. Figure 4b represents the microstructure of Al–20%Si alloy with addition of 0.4% Al–5Ti–C alloy. It can be clearly observed that the size and inter-flake spacing of eutectic Si significantly decreases with the addition of Al–5Ti–C alloy. Moreover, the coarse acicular and plate-like eutectic Si structure was transformed into a branched morphology, and the edges and corners of the eutectic Si became smooth and round. Figure 4c shows the microstructure of eutectic Si in Al–20%Si alloy with the addition of 0.6% Al–5Ti–C alloy. It is apparent that most of the eutectic Si structure changed to a fine fibrous structure with discrete particles. However when the addition of Al–5Ti–C alloy was further increased to 1.0%, 1.6% and 2.0%, the average size of the eutectic Si crystals increased and the morphology of the eutectic Si structure was transformed into a coral-like fibrous structure with coarse needles and platelets, as shown in Figure 4d,f.

**Figure 5.**
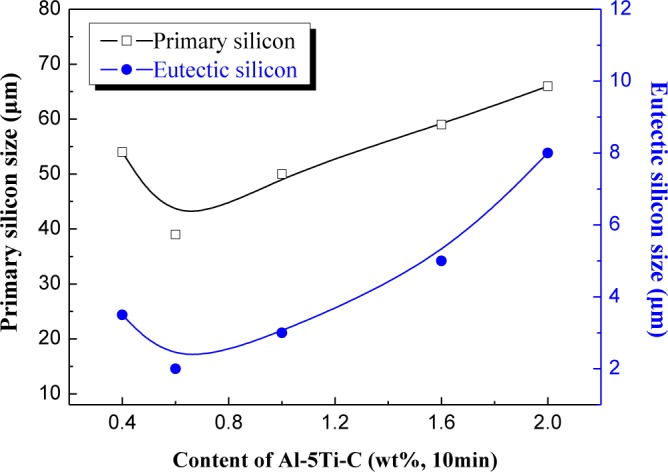
The relation curve between the content of Al–5Ti–C and the average size of primary Si and eutectic Si in hypereutectic Al–20%Si alloy.

Figure 5 shows the relation curve between the content of Al–5Ti–C master alloy and the size of primary Si and eutectic Si of Al–20%Si alloys. It can be seen that when the addition amount of Al–5Ti–C master alloy is less than 0.6 wt%, the primary Si and eutectic Si in the hypereutectic Al–20%Si alloy is in the less deterioration state; when the amount of Al–5Ti–C master alloy added is more than 0.6 wt%, the primary Si and eutectic Si in the hypereutectic Al–20%Si alloy is in the over deterioration state; with regard to less deterioration, we think it is due to an insufficient amount of modifier added. For over deterioration, it may be that the TiC and TiAl_3_ added in the alloy melt interact with other alloying elements in the melt and form new compounds, so that the effective content of modifier in the alloy melt is decreased, which finally results in inadequate deterioration. The results show that 0.6% Al–5Ti–C alloy cannot only effectively decrease the size of primary Si and eutectic Si crystals in hypereutectic Al–20%Si alloy, but it can also change the morphology of primary Si and eutectic Si crystals.

**Figure 6.**
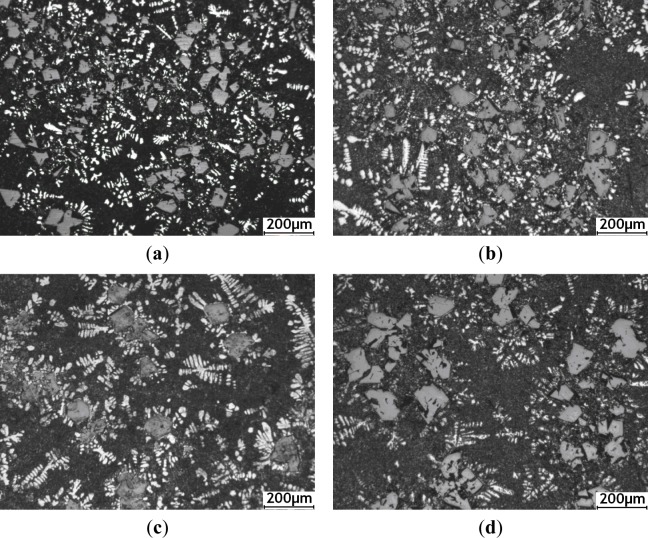
Optical micrographs of primary Si crystals in hypereutectic Al–20%Si alloy with 0.6 wt% Al–5Ti–C master alloy at different heat treatment times: (**a**) 30 min; (**b**) 60 min; (**c**) 90 min; and (**d**) 120 min.

**Figure 7.**
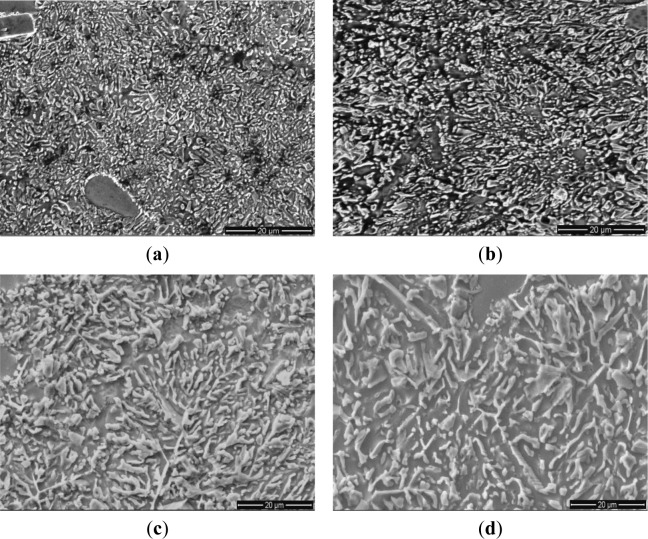
SEM images of eutectic Si crystals in hypereutectic Al–20%Si alloy with 0.6 wt% Al–5Ti–C master alloy at different heat treatment times: (**a**) 30 min; (**b**) 60 min; (**c**) 90 min; and (**d**) 120 min.

**Figure 8.**
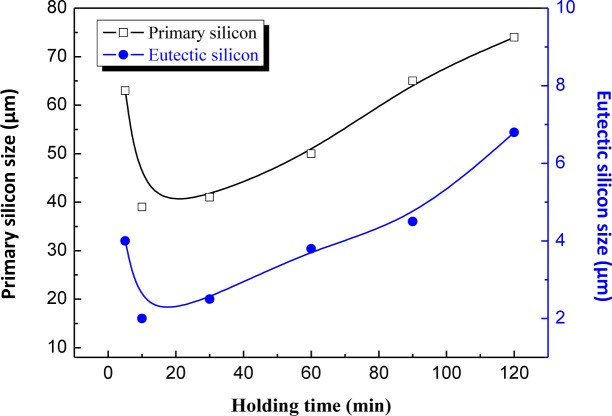
The relation curve between holding time and the size of primary Si and eutectic Si in hypereutectic Al–20%Si alloy with 0.6 wt% Al–5Ti–C master alloy.

Figures 6 and 7 show the microstructures of the Al–20% Si alloy in which Al–5Ti–C master alloy, whose mass fraction is 0.6%, is added and is then heat preserved for different times. As can be seen, the deterioration effect of Al–5Ti–C master alloy on the primary Si and eutectic Si in the hypereutectic Al–20% Si alloy varies with the heat treatment time. Figure 8 shows the relationship curve between the average size of the primary Si and eutectic Si in the hypereutectic Al–20 wt% Si alloy and different heat treatment times of refinement and deterioration. It can be seen that when the heat treatment time is 30 min, the average size of primary Si is roughly 41 μm (Figure 6a), and the size of eutectic Si is roughly 2–3 μm (Figure 7a). With the increase in heat treatment time of refinement and deterioration, the sizes of primary Si and eutectic Si in the alloy increase gradually. When the heat treatment time is 120 min, the morphology of primary Si is again changed into a coarse and complex picture (Figure 6d), while the eutectic Si is changed from ball-shaped or short-rod-like structures into long needle-like ones (Figure 7d).

Barerji and Reif [27] from Berlin University indicated that since TiAl_3_ is thermodynamically unstable, it will be dissolved in liquid aluminum at a speed of 40 μm/min. López experimentally found that when the temperature of Al–Si alloy is higher than 600 °C, the TiC in the alloy decomposes, and decomposition of TiC in the Al–Si alloy is achieved through continuous diffusion of Ti and C into the alloy [28]. In this experiment, when Al–5Ti–C master alloy was added into hypereutectic Al–20 wt% Si alloy, after being heat preserved for 10 min, the full decomposition of TiAl_3_ and TiC particles occurred, which thus provides sufficient amount of Ti atoms for the aluminum melt. The dissolution of TiAl_3_ causes an equilibrium of dissolved Ti in the bulk melt. It results in the establishment of an activity gradient which leads to solute segregation, and the well-known contact time in grain refining practice is a result of this process [29]. Whenever an element is exposed to an activity gradient, there is a tendency to undergo net transport [30].Thus, Ti atoms congregate at the growing surface of the primary Si and eutectic Si based on the activity gradient of Ti. During the solidification of the alloy melt, the Ti-rich zone is easy to form on the growing Si surface. The Ti-rich layer will prevent the Si atoms in the melt from diffusing toward the growing surface of primary Si and eutectic Si, thereby inhibiting the growth of primary Si and eutectic Si, and thus indirectly refining them. However, as the heat treatment time increases, when Ti atoms concentrate to a certain degree in these areas and exceed their saturation value, the Ti-rich layer will change into TiAl_3_ [31,32], thus losing the inhibition effect of primary Si and eutectic Si, and leading to recession of the refinement and deterioration effect. The refinement mechanism of Al alloy is extremely complicated, and it is also very difficult to fully analyze the refinement process and mechanism of Al-Ti-C toward Al–20%Si alloy.

### 2.3. Analyses of Mechanical Properties

**Figure 9.**
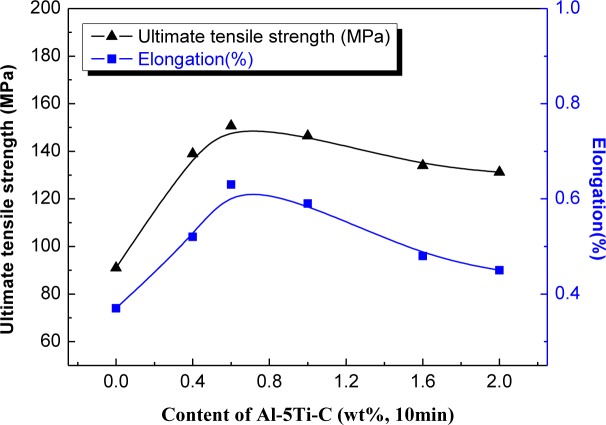
Mechanical properties of hypereutectic Al–20%Si alloy with various contents of Al–5Ti–C master alloy.

Figure 9 shows typical ultimate tensile strength (UTS) and elongation (El) with different levels of Al–5Ti–C master alloy. It is evident that the ultimate tensile strength (UTS) is enhanced by roughly 65% from 91 to 150 MPa, and elongation is increased by roughly 70% from 0.37% to 0.63% after the addition of 0.6% Al–5Ti–C master alloy. A further increase in the amount of addition of Al–5Ti–C master alloy up to 1.0% leads to a decrease in both UTS and El. The results are in agreement with the aforementioned images (Figures 3 and 4). The mechanical properties of hypereutectic Al–20%Si alloy mainly depend on the morphology and size of primary Si and eutectic Si crystals. It has been reported that the cracks easily initiate from the stiff and brittle primary Si as well as eutectic Si crystals [3]. On the other hand, the cracks may come from debonding of Si particles, and then propagate through the boundaries with the α-Al phase [15]. Therefore, both UTS and El significantly improve when primary and eutectic Si phases are modified by adding Al–5Ti–C master alloy due to decreasing or eliminating premature crack initiation and fracture in tension.

**Figure 10.**
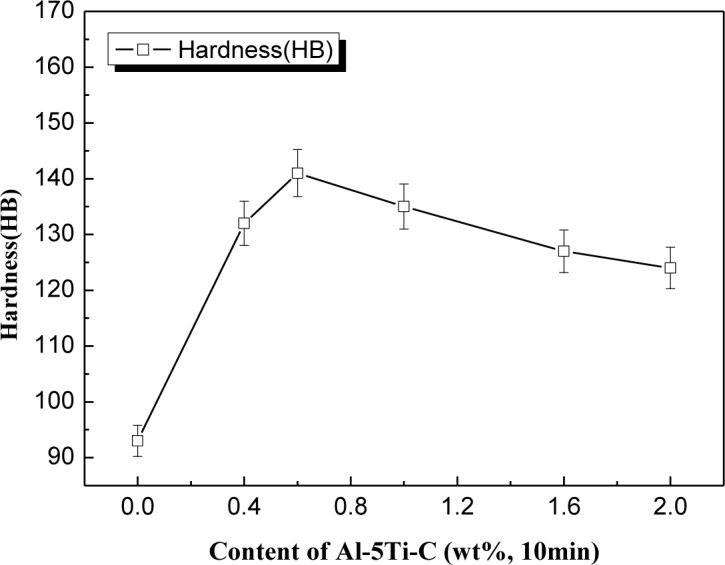
Brinell hardness of hypereutectic Al–20%Si alloy with various contents of Al–5Ti–C master alloy.

Figure 10 shows that the ingot cast without any mould coating has the lowest hardness value. However, as Al–5Ti–C master alloy was introduced into the mould coating and its concentrations progressively increased from 0 to 0.6 wt%, the hardness value increased by roughly 51% from 93 to 141 HB. Above 0.6 wt% Al–5Ti–C master alloy concentration the hardness value decreased. This shows that the optimum value of Al–5Ti–C master alloy in alumina based mould wash which gives us the maximum hardness value (HB141) is 0.6 wt%. It is expected that the hardness may depend on the grain size of the Si phase following the Hall-Petch type equation [33,34]:

$$H=H_{0}+K_{H}D^{-\frac{1}{2}}$$ (1)

where *H* is the hardness, *H*_0_ is the hardness expected at a hypothetical infinite grain size, *K_H_* is a constant, and *D* is the average grain diameter. The result causes the Brinell hardness of the modified Al–Si alloys to be higher than that of the unmodified Al–20%Si alloys.

## 3. Experimental Section

The main materials used in the experiments included: Al powder (99.6%), Ti powder (99.3%), C powder (99.8%) and commercially pure aluminum. The main raw materials were made through ball mixing and cold pressing into prefabricated blocks. The prefabricated blocks went through a thermal explosion reaction in the pure molten aluminum at a temperature of 780 °C [35]. Analysis was conducted on the phase composition, microstructure morphology and components of the alloy with RigakuD/max–A X-ray diffract meter (XRD, PW 3040/60, PANalytical, Rotterdam, Holland), large optical microscope (MEF3) and the JSM–7500 scanning electron microscope (SEM, SSX–550 fitted with EDS equipment, Shimadzu Corporation, Kyoto, Japan).

The 0.5 kg hypereutectic Al–20%Si (all percentages are wt% unless otherwise stated) alloy used in the experiment was melted in an electrical resistance furnace. The melt was brought to a temperature of 750 °C and degassed using a commercial degasser of solid hexachloroethane (C_2_Cl_6_). Subsequently, the different additions of Al–5Ti–C master alloy (0.4%, 0.6%, 1.0%, 1.6% and 2.0%) were added to the alloy at 750 °C, stirred and held for 10 min to ensure homogeneity of composition. Al–5Ti–C master alloy (0.6 wt%) was added to the alloy, stirred and held for 30, 60, 90 and 120 min at 750 °C. After the slag of the melt was skimmed, the melt was poured into a preheated (about 200 °C) permanent steel mould (20 mm in inner diameter and 50 mm in length) at 720 °C. Microstructural survey of samples was conducted with optical microscopy (OM) and scanning electron microscopy (SEM) after preparing samples according to standard metallographic procedures, which were etched using Keller’s reagent (2.5 mL HNO_3_, 1.5 mL HCl, 1 mL HF, and 95 mL H_2_O).The unmodified ingot was obtained under the same procedure without adding Al–5Ti–C master alloy. The average value of Brinell hardness was taken and tensile tests were carried out on MTS810 from five measurements.

## 4. Conclusions

Al–5Ti–C master alloy was prepared and the effect of different quantities of Al–5Ti–C master alloy on the microstructure and mechanical properties of Al–20%Si alloy was studied. The following conclusions were drawn:

(i) Al–5Ti–C master alloy with a more uniform microstructure was successfully prepared through a method of liquid solidification reactions, composed of Al, TiAl_3_ and TiC.
(ii) Al–5Ti–C master alloy can significantly refine primary Si crystals such that their sizes decrease from roughly 90–120 to 20–50 μm and the morphology changes from a coarse star-like and polygonal shape to a fine blocky shape at a concentration of 0.6% Al–5Ti–C alloy while being held for 10 min.
(iii) Al–5Ti–C master alloy can obviously modify the eutectic Si structure and cause a transition from the coarse flake-like and acicular shape to a fine fibrous structure with discrete particles at a concentration of 0.6% Al–5Ti–C alloy while being held for 10 min.
(iv) When the concentration of Al–5Ti–C master alloy is 0.6%, with increasing heat treatment time up to 30 min, the Al–5Ti–C master alloy still undergoes a good modified effect.
(v) Due to the refinement and modification of primary and eutectic Si crystals, the ultimate tensile strength (UTS), elongation (El) and Brinell hardness (HB) are significantly improved on increasing the concentration of Al–5Ti–C master alloy up to 0.6%, in which the ultimate tensile strength increases by roughly 65%, the elongation increases by roughly 70% and the Brinell hardness increases by roughly 51%.

## Figures and Tables

**Table 1.** EDS composition analysis of point A and point B in Figure 2.

Point No.	*x*(Al)/%	*x*(Ti)/%	*x*(C)/%
A	69.72	21.85	8.43
B	46.50	25.52	27.98
